# Intensity standardization methods in magnetic resonance imaging of head and neck cancer

**DOI:** 10.1016/j.phro.2021.11.001

**Published:** 2021-11-20

**Authors:** Kareem A. Wahid, Renjie He, Brigid A. McDonald, Brian M. Anderson, Travis Salzillo, Sam Mulder, Jarey Wang, Christina Setareh Sharafi, Lance A. McCoy, Mohamed A. Naser, Sara Ahmed, Keith L. Sanders, Abdallah S.R. Mohamed, Yao Ding, Jihong Wang, Kate Hutcheson, Stephen Y. Lai, Clifton D. Fuller, Lisanne V. van Dijk

**Affiliations:** a Departments of Radiation Oncology, The University of Texas MD Anderson Cancer Center, Houston, TX, United States; b Imaging Physics, The University of Texas MD Anderson Cancer Center, Houston, TX, United States; c Radiation Physics, The University of Texas MD Anderson Cancer Center, Houston, TX, United States; d Head and Neck Surgery, The University of Texas MD Anderson Cancer Center, Houston, TX, United States

**Keywords:** MRI, Standardization, Harmonization, Normalization, Quantitative analysis, Head and neck cancer

## Abstract

**Background and Purpose:**

Conventional magnetic resonance imaging (MRI) poses challenges in quantitative analysis because voxel intensity values lack physical meaning. While intensity standardization methods exist, their effects on head and neck MRI have not been investigated. We developed a workflow based on healthy tissue region of interest (ROI) analysis to determine intensity consistency within a patient cohort. Through this workflow, we systematically evaluated intensity standardization methods for MRI of head and neck cancer (HNC) patients.

**Materials and Methods:**

Two HNC cohorts (30 patients total) were retrospectively analyzed. One cohort was imaged with heterogenous acquisition parameters (HET cohort), whereas the other was imaged with homogenous acquisition parameters (HOM cohort). The standard deviation of cohort-level normalized mean intensity (SD NMI_c_), a metric of intensity consistency, was calculated across ROIs to determine the effect of five intensity standardization methods on T2-weighted images. For each cohort, a Friedman test followed by a post-hoc Bonferroni-corrected Wilcoxon signed-rank test was conducted to compare SD NMI_c_ among methods.

**Results:**

Consistency (SD NMI_c_ across ROIs) between unstandardized images was substantially more impaired in the HET cohort (0.29 ± 0.08) than in the HOM cohort (0.15 ± 0.03). Consequently, corrected p-values for intensity standardization methods with lower SD NMI_c_ compared to unstandardized images were significant in the HET cohort (p < 0.05) but not significant in the HOM cohort (p > 0.05). In both cohorts, differences between methods were often minimal and nonsignificant.

**Conclusions:**

Our findings stress the importance of intensity standardization, either through the utilization of uniform acquisition parameters or specific intensity standardization methods, and the need for testing intensity consistency before performing quantitative analysis of HNC MRI.

## 1 Introduction

Magnetic resonance imaging (MRI) is routinely used in clinical practice and has revolutionized how physicians evaluate diseases [1]. Conventional “weighted” MRI, where various acquisition parameters are modulated to generate T1-weighted (T1-w) or T2-weighted (T2-w) images, has become commonplace in clinical workflows. Although conventional MRI acquisitions are useful for the qualitative assessment of disease, advanced quantitative evaluation (e.g., through radiomics [2] or deep learning [3]) is seemingly precluded by a fundamental problem: arbitrary voxel intensity. Unlike computed tomography, in which voxel intensities correspond to inherent tissue properties, the absolute voxel intensities of MRI correspond to both tissue properties and hardware-specific settings [4] and thus do not have a specific physical meaning. Consequently, MRI voxel intensity can vary from scanner to scanner and even within the same scanner [5]. A few important exceptions include images generated through various quantitative MRI acquisitions [6], such as diffusion-weighted MRI [7], dynamic contrast-enhanced MRI [8], or T1/T2 mappings [9], which are not routinely acquired in standard-of-care imaging. Unfortunately, intensity standardization (sometimes referred to as normalization or harmonization) is an often overlooked but crucial pre-processing step in studies attempting a quantitative analysis of conventional MRI acquisitions.

MRI is often performed for head and neck cancer (HNC) patients as part of radiotherapy treatment planning. Weighted images, particularly T2-w images, are commonly acquired in the scanning protocol because they provide excellent soft-tissue contrast in the complex anatomical areas involved in HNC. Thus, they are useful for region-of-interest (ROI) delineation [10], [11]. Notably, the increasing use of MRI-guided technology for adaptive HNC radiotherapy will likely increase the clinical integration of MRI quantitative analysis [12]. While several recent HNC studies have implemented cohort-level quantitative analysis of conventional weighted MRI [13], [14], [15], [16], [17], [18], [19], [20], [21], [22], relatively few have investigated incorporating intensity standardization into processing pipelines [16], [17], [18], [19], [20], [22], and even fewer have tested multiple standardization methods [20]. Furthermore, while rigorous studies have tested MRI intensity standardization methods for various anatomical regions, chiefly the brain [5], [23], such methods for the head and neck region have yet to be systematically investigated. The head and neck region may pose additional challenges for MRI intensity standardization when compared with relatively piecewise homogeneous regions like the brain. For instance, fields of view often vary across acquisitions, and the head and neck region is home to many tissue-tissue and tissue-air interfaces [24], which may result in a greater range and complexity of the underlying intensity distributions. Therefore, there is a pressing need to systematically investigate the effects of available MRI intensity standardization methods in HNC cohorts.

To address the growing importance of intensity standardization in the quantitative analysis of HNC MRI, we developed a novel, ROI-based workflow to compare existing standardization methods in T2-w images of HNC patients. We used two independent HNC cohorts—a multi-institutional cohort with heterogeneous acquisition parameters and a single-institutional cohort with homogeneous acquisition parameters—to systematically determine the effect of different intensity standardization methods for HNC MRI.

## 2 Materials and methods

### 2.1 Patient cohorts, image acquisitions, and ROIs

Two separate sets of patients who were diagnosed with oral or oropharyngeal cancer and for whom T2-w MRI images were available before the start of radiotherapy were included in this proof-of-concept study. A subset (cohort) of patients from each set was randomly selected for the analysis. The first cohort consisted of 15 patients with images acquired at different institutions and was termed “heterogeneous” (HET) because of the variety of scanners and acquisition parameters used in image generation. All 15 patients in the HET cohort were imaged with different scanning protocols; the MRI scanners originated from several manufacturers, including Siemens, GE, Phillips, and Hitachi. The second cohort consisted of 15 patients from a single prospective clinical trial with the same imaging protocol (NCT03145077, PA16-0302) and was termed “homogeneous” (HOM) because of the uniformity of both the scanner and the acquisition parameters used for image generation. Patients in the HOM cohort were imaged on a Siemens Aera scanner and immobilized with a thermoplastic mask. Image acquisition parameters for the cohorts are shown in Table 1; the demographic characteristics of each cohort are summarized in Table 2. All images were retrospectively collected in the Digital Imaging and Communications in Medicine (DICOM) format under a HIPAA-compliant protocol approved by our institution's IRB (RCR03-0800). The protocol included a waiver of informed consent. The anonymized image sets analyzed during the current study are publicly available online through Figshare (https://doi.org/10.6084/m9.figshare.13525481). For each image, ROIs of various healthy tissue types and anatomical locations were manually contoured in the same relative area for five slices by one observer (medical student) using Velocity AI v.3.0.1 (Atlanta, GA, USA), verified by a physician expert (radiologist), and exported as DICOM-RT Structure Set files. The ROIs were: 1. cerebrospinal fluid inferior (CSF_inf), 2. cerebrospinal fluid middle (CSF_mid), 3. cerebrospinal fluid superior (CSF_sup), 4. cheek fat left (Fat_L), 5. cheek fat right (Fat_R), 6. nape fat inferior (NapeFat_inf), 7. nape fat middle (NapeFat_mid), 8. nape fat superior (NapeFat_sup), 9. neck fat (NeckFat), 10. masseter left (Masseter_L), 11. masseter right (Masseter_R), 12. rectus capitus posterior major (RCPM), 13. skull, and 14. cerebellum. A visual representation of the ROIs is shown in Supplementary Figure S4. All DICOM and radiotherapy structure files were converted to Python data structures for processing and analysis with DICOMRTTool v.0.3.21 [25].

**Table 1:** MRI acquisition parameters for heterogeneous (HET) and homogeneous (HOM) cohorts.*

MRI Acquisition Parameter	HET Cohort (n = 15)	HOM Cohort (n = 15)
Magnetic Field Strength (T)	1.50–3.00	1.50
Repetition Time (ms)	3000.00–8735.00	4800.00
Echo Time (ms)	70.80–123.60	80.00
Echo Train Length	10–65	15
Flip Angle (°)	90–150	180
In-plane Resolution (mm)	0.35–1.01	0.50
Slice Thickness (mm)	2.00–6.00	2.00
Spacing Between Slices (mm)	1.00–7.00	2.00
Imaging Frequency (MHz)	12.68–127.77	63.67
Number of Averages	1.00–4.00	1.00
Percent Sampling (%)	78.91–100	90.00

*Data shown for HET cohort are ranges. All HOM cohort patients had the same scanning parameters.

**Table 2:** Patient demographic characteristics for heterogeneous (HET) and homogeneous (HOM) cohorts.*

Characteristic	HET Cohort (n = 15)	HOM Cohort (n = 15)
Age (median, range)	61 (41–78)	61 (46–77)
Patient Sex		
Men	13	14
Women	2	1
T Stage		
T1	5	7
T2	6	3
T3	1	1
T4	3	4
N Stage		
N0	4	0
N1	5	4
N2	6	11
Primary Tumor Site		
Base of Tongue	5	9
Tonsil	6	6
Oral Cavity	4	0

*Unless otherwise indicated, data shown are number of patients.

### 2.2 Intensity standardization methods

We applied a variety of MRI intensity standardization methods to both cohorts’ images. These methods were chosen because of their relative ubiquity in other studies and simple implementations. Details of the implementation of these methods are presented below.

1. Unstandardized (*Original*)*:* No intensity standardization was performed.

2. Rescaling (*MinMax*)*:* This method standardized the image by rescaling the range of values to [0,1] using the equation

$$f\left(x\right)=\frac{x-min(x)}{\max(x)-min(x)}$$

where *x* and *f(x)* were the original and standardized intensities, respectively, and min*(x)* and max*(x)* were the minimum and maximum image intensity values per patient, respectively.

3. Z-score standardization using all voxels (*Z-All*)*:* This method standardized the image by centering it at a mean of 0 with a standard deviation of 1. The standardization was based on all voxels in the image and used the equation

$$f\left(x\right)=\frac{x-\mu_{x}}{\sigma_{x}}$$

where *x* and *f(x)* were the original and standardized voxel intensities, respectively, and *μ_x_* and *σ_x_* were the mean and standard deviation of the image intensity values per patient, respectively.

4. Z-score standardization using only voxels in an external mask (*Z-External*)*: Z-All* was performed as described in item 3 above, but *μ_x_* and *σ_x_* were derived from voxels located in an external mask of the head and neck region (Supplementary Figure S5).

5. Cheek fat standardization (*Fat*): This method standardized the image with respect to left and right cheek fat (healthy tissue) and was adapted from van Dijk et al. [19]. The intensity of each voxel was divided by the mean intensity of the cheek fats and multiplied by an arbitrary scaling value of 350 using the equation

$$f\left(x\right)=\frac{x}{\mu_{\mathrm{fat}}}*350$$

where *x* and *f(x)* were the original and standardized intensities, respectively, and *μ_fat_* was the mean intensity of both cheek fat ROIs per patient.

6. Histogram standardization (*Nyul*)*:* This method was adapted from Nyul and Udupa [26] using a code implementation from Reinhold et al. [27]. It used images for all patients in a cohort to construct a standard histogram template through the determination of histogram parameters and then linearly mapped the intensities of each image to the standard histogram template. The histogram parameters in this implementation were defined as intensity percentiles at 1, 10, 20, 30, 40, 50, 60, 70, 80, 90, and 99 percent. Only voxels within the head and neck external mask were used in the construction of the standard histogram template.

### 2.3 Intensity-Based ROI evaluation

According to the statistical principles of image normalization criteria [5], MRI intensities for a single type of tissue should maintain similar distributions within and across patients. Therefore, for a set of nonpathological ROIs representing a corresponding set of tissues within a cohort of patients, an increase in the quality of MRI intensity standardization should lead to an increase in the consistency of ROI intensity distributions (Supplementary Figure S6). Importantly, our aim is not to match distributions of the entire image since targets of quantitative analysis (e.g., tumors or healthy tissues altered by radiotherapy, such as the parotid glands) are expected to vary among patients. Motivated by this goal of population-level analysis that relies on the consistency of replicable units within tissue types and across patients, we used a simple and interpretable metric of comparison to quantify ROI intensity histogram overlap—termed the standard deviation of cohort-level normalized mean intensity (SD NMI_c_)—which can be applied before or after an intensity standardization procedure in a given cohort. The steps to calculate this metric are provided in Supplementary Data S1. Briefly, the metric is calculated by dividing the mean of a given ROI intensity distribution for a patient by the range of distributions for a cohort and then computing the standard deviation of the resulting values for the entire cohort. Given a set of ROIs that are not anticipated to vary from patient to patient, we would expect that for an ideal intensity standardization method, the cumulative SD NMI_c_ would remain close to 0. For both cohorts, the SD NMI_c_ was calculated for each intensity standardization method per ROI and visually compared on a heatmap. Of note, the cheek fat ROIs were not included in the evaluation since they were used for the *Fat* standardization method, and this could bias results.

### 2.4 Statistical analysis

After applying the Shapiro-Wilk test for normality [28], we found the SD NMI_c_ to be non-normally distributed (p < 0.05). Therefore, nonparametric tests were deemed appropriate for statistical analysis. For each cohort, the Friedman test [29], a nonparametric analog to the one-way repeat measures analysis of variance test, was conducted to compare SD NMI_c_ values among intensity standardization methods with standardization methods acting as within-subject factors. We note that the Friedman test included unstandardized (*Original*) images. If the Friedman test was statistically significant, a subsequent post-hoc two-sided Wilcoxon signed-rank test with a Bonferroni correction [30] was performed for all pair-wise combinations of standardization methods to determine which methods were significantly better than others. For both the Friedman and Wilcoxon signed-rank tests, p-values<0.05 were considered significant. Statistical analysis was performed in Python v.3.7.6. The code used to produce our analysis is available through GitHub (https://github.com/kwahid/MRI_Intensity_Standardization). The overall workflow of our approach is shown in Supplementary Figure S7.

## 3 Results

**Fig. 1:**
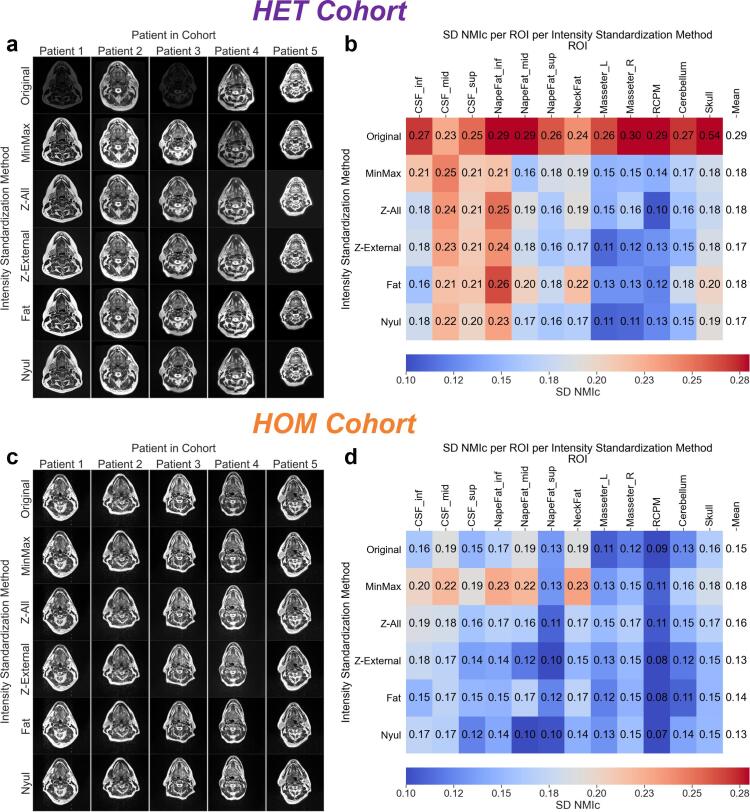
Intensity standardization comparisons for the heterogeneous (HET) and homogeneous (HOM) cohorts. Single-slice representations of T2-weighted images from each intensity standardization method for five patients from the **(a)** HET and **(c)** HOM cohorts. Images for each method in each cohort are displayed using the same window width and center. Standard deviation of cohort-level normalized mean intensity (SD NMI_c_) heatmaps of intensity standardization methods by region of interest (ROI) for **(b)** HET and **(d)** HOM cohorts. The resulting means across all ROIs for each method are shown in the rightmost columns of the heatmaps.

On visual inspection, unstandardized images from the HET cohort had noticeably different intensities when compared to standardized images (Fig. 1**a**). These qualitative observations were confirmed through quantitative estimates of intensity consistency where unstandardized images compared to standardized images often had higher SD NMI_c_ values in the HET cohort (Fig. 1**c**). Oppositely, in the HOM cohort unstandardized images were similar to standardized images qualitatively (Fig. 1**b**) and quantitatively (Fig. 1**d**). When considering SD NMI_c_ across all tissue sites (mean ± SD), the worst method was *Original* (0.29 ± 0.08) in the HET cohort and *MinMax* (0.18 ± 0.04) in the HOM cohort. Conversely, the best method was a tie between *Z-External* and *Nyul* for both cohorts, where SD NMI_c_ was 0.17 ± 0.04 in the HET cohort and 0.13 ± 0.03 in the HOM cohort. Additional intermediary data in the form of ROI intensity distributions for the HET and HOM cohorts are found in Supplementary Figure S8 and Supplementary Figure S9, respectively, and demonstrated consistency with the SD NMI_c_ values presented. Moreover, we performed additional analysis on T1-weighted images in a subset of cases from the HOM cohort in Supplementary Data S2, and demonstrated similar results to our observations on T2-weighted images.

**Fig. 2:**
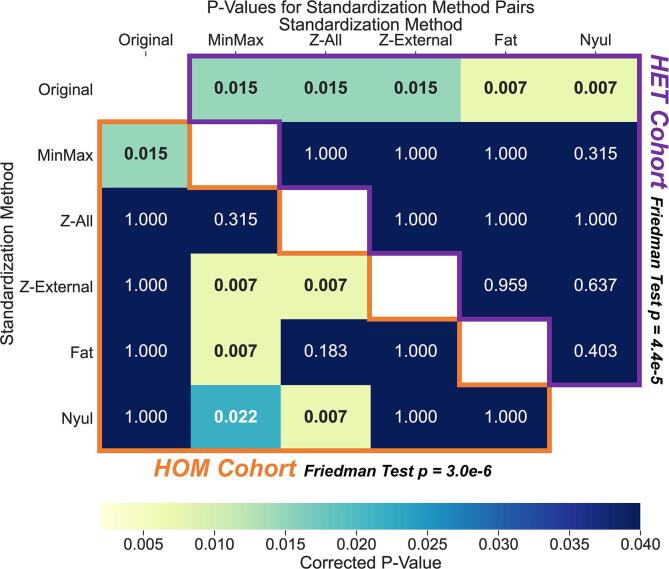
Statistical comparison matrix of standard deviation of cohort-level normalized mean intensity (SD NMI_c_) values between the intensity standardization methods for the heterogeneous (HET) and homogeneous (HOM) cohorts. Freidman test results are shown adjacent to the cohort titles. Each matrix entry corresponds to a corrected p-value for a standardization method pair resulting from a two-sided Wilcoxon signed-rank test between SD NMI_c_ values for each healthy tissue region of interest. Significant values (p < 0.05) are in bold in the matrix. The HOM cohort results are outlined in orange below the white diagonal entries, whereas the HET cohort results are outlined in purple above the white diagonal entries. (For interpretation of the references to colour in this figure legend, the reader is referred to the web version of this article.)

The Friedman tests showed that SD NMI_c_ values across all ROIs were significantly different for the intensity standardization methods in both cohorts (p < 0.001) (Fig. 2). Post-hoc analysis in the HET cohort (Fig. 2, above diagonal) revealed significantly higher SD NMI_c_ values in *Original* compared with *MinMax* (p < 0.05), *Z-All* (p < 0.05), *Z-External* (p < 0.05), *Fat* (p < 0.01), and *Nyul* (p < 0.01). None of the intensity standardization methods in the HET cohort had significantly different SD NMI_c_ values when compared with each other (p > 0.05). Post-hoc analysis in the HOM cohort (Fig. 2, below diagonal) revealed significantly lower SD NMI_c_ values in *Original* compared with *MinMax* (p < 0.05). None of the other standardization methods in the HOM cohort (*Z-All*, *Z-External*, *Fat*, and *Nyul*) had significantly different SD NMI_c_ values compared with *Original* (p > 0.05). Moreover, the HOM cohort demonstrated significantly higher SD NMI_c_ values in *MinMax* compared with *Z-External* (p < 0.01), *Fat* (p < 0.01), and *Nyul* (p < 0.05). Finally, the HOM cohort demonstrated significantly higher SD NMI_c_ values in *Z-All* when compared with *Z-External* (p < 0.01), and *Nyul* (p < 0.01).

## 4 Discussion

In this study, we proposed a workflow to test the consistency of standardized and unstandardized conventional MRI within a given HNC cohort. The scale-invariant, and thus comparable, SD NMI_c_ metric was calculated to systematically investigate the effects of intensity standardization methods on T2-w images for two independent cohorts of HNC patients based on healthy tissue intensity consistency in multiple ROIs. Broadly, we determine that depending on the underlying imaging characteristics of a given cohort, explicit intensity standardization has varying effects on intensity consistency, which has the potential to impact downstream quantitative analysis.

Our results show that intensity standardization, when compared to no standardization, substantially improved T2-w MRI ROI intensity consistency in the HET cohort (Fig. 1**a,b**), but had a minimal impact in the HOM cohort (Fig. 1**c,d**). Since scanner and acquisition parameters were vastly different in the HET cohort, the marked intensity variation between *Original* images (Fig. 1**a**) was in line with our expectations. In contrast, while the HOM cohort included patients from clinical trial data with the same scanner and acquisition parameters, the relatively minor intensity variation between *Original* images (Fig. 1**c**) was better than expected. In a sense, the use of identical acquisition parameters in the HOM cohort seemed to act as an inherent method of pre-processing intensity standardization. This may also indicate that flexible head and neck MRI coil positioning was performed systematically and reliably in this cohort, as positioning the coil at varying distances from the patient can result in different image intensities. While the use of uniform acquisition parameters on the same MRI scanner may circumvent the need for the application of intensity standardization methods, further work is likely needed to verify these results.

Upon visual inspection, it was difficult to discriminate between the various intensity standardization methods for either cohort (Fig. 1**a,c**). Quantitatively, most intensity standardization methods had similar performance in both cohorts, regardless of overall consistency improvement compared to unstandardized images (Fig. 1**b,d** and Fig. 2). Recent work by Carré et al [31]. demonstrated results similar to those of our study in that various intensity standardization methods improved the consistency between brain images with heterogeneous acquisition parameters, although the authors did not identify a specific superior standardization method. In the current study, paired significance testing (Fig. 2) revealed that the standardization methods *Z-External* and *Nyul* performed relatively well in both the HET cohort (significantly better than *Original*) and the HOM cohort (significantly better than *MinMax* and *Z-All*), with both methods achieving the lowest SD NMI_c_ values. Interestingly, the only methods that performed significantly worse than any others in the HOM cohort were *MinMax* and *Z-All*, possibly secondary to the large number of background elements influencing the standardization parameters in the HOM cohort.

A potential limitation of this proof-of-concept study was that it included a small number of patients for each cohort. However, we implemented conservative significance testing to ensure the robustness of our results. Moreover, this study focused on analyzing T2-w sequences for initial testing since they are favored in head and neck imaging due to their exquisite anatomical detail for various ROIs [32], [33]. Since healthy tissue ROIs are regularly contoured during radiotherapy treatment planning, our analysis tools and workflow can facilitate future large-scale HNC investigations with additional MRI sequences. To guide further research in different MRI sequences, we present preliminary analyses on T1-weighted images from the HOM cohort in Supplementary Data S2, which are broadly consistent with the findings in this study. Another limitation of our study is that our workflow may not apply to patients who have already received radiotherapy, as radiation can cause structural and functional changes in healthy tissue that may impact the intensity profiles of various ROIs [34]. This may be mitigated by selecting ROIs that are known not to change significantly with treatment. Finally, acquisition artifacts, such as magnetic field inhomogeneities, can affect MRI intensity [35]. While images from the HOM cohort were free of artifacts, some images from the HET cohort contained bias fields (Supplementary Figure S10). Therefore, bias-field correction techniques may need to be explored in combination with intensity standardization methods in future studies to determine their effects on ROI intensity consistency in HNC MRI.

To our knowledge, this is the first study to systematically investigate the effects of intensity standardization in head and neck imaging. In addition, whereas many studies of MRI intensity standardization for other anatomical sites implemented test–retest data for individual patients to determine the effects of standardization [31], [36], [37], [38], [39], our analysis is unique because it investigated the impact of standardization within a cohort of patients. Our approach may be more relevant to the downstream cohort-level model construction often implemented in quantitative analysis studies. Finally, we have publicly disseminated our data and analysis tools through open access platforms to foster increased reproducibility and help the imaging research community extrapolate our workflow to MRI of other anatomical regions.

In summary, we propose a workflow to robustly test MRI intensity consistency within a given HNC patient cohort and demonstrate the need for evaluating MRI intensity consistency before performing quantitative analysis. This study verifies that intensity standardization, either through the utilization of uniform acquisition parameters or specific intensity standardization methods, is crucial to improving the consistency of inherent tissue intensity values in conventional weighted HNC MRI. Our study is an essential first step towards widespread intensity standardization for quantitative analysis of conventional MRI in the head and neck region.

## Funding Statement

This work was supported by the National Institutes of Health (NIH) through a Cancer Center Support Grant (P30-CA016672-44). K.A. Wahid and T. Salzillo are supported by training fellowships from The University of Texas Health Science Center at Houston Center for Clinical and Translational Sciences TL1 Program (TL1TR003169). K.A. Wahid is also supported by the American Legion Auxiliary Fellowship in Cancer Research and an NIDCR F31 fellowship (1 F31 DE031502-01). R. He., A.S.R. Mohamed, K. Hutcheson, and S.Y. Lai are supported by a NIH National Institute of Dental and Craniofacial Research (NIDCR) Award (R01DE025248). B.A. McDonald receives research support from an NIH NIDCR Award (F31DE029093) and the Dr. John J. Kopchick Fellowship through The University of Texas MD Anderson UTHealth Graduate School of Biomedical Sciences. B.M. Anderson receives funding from a Society of Interventional Radiology Foundation Allied Scientist Grant and the Dr. John J Kopchick Fellowship. L.A. McCoy and K.L. Sanders are supported by NIH NIDCR Research Supplements to Promote Diversity in Health-Related Research (R01DE025248-S02 and R01DE028290-S01 respectively). M.A. Naser and S. Ahmed are supported by a NIH NIDCR Award (R01 DE028290-01). C.D. Fuller received funding from an NIH NIDCR Award (1R01DE025248-01/R56DE025248) and Academic-Industrial Partnership Award (R01 DE028290), the National Science Foundation (NSF), Division of Mathematical Sciences, Joint NIH/NSF Initiative on Quantitative Approaches to Biomedical Big Data (QuBBD) Grant (NSF 1557679), the NIH Big Data to Knowledge (BD2K) Program of the National Cancer Institute (NCI) Early Stage Development of Technologies in Biomedical Computing, Informatics, and Big Data Science Award (1R01CA214825), the NCI Early Phase Clinical Trials in Imaging and Image-Guided Interventions Program (1R01CA218148), the NIH/NCI Cancer Center Support Grant (CCSG) Pilot Research Program Award from the UT MD Anderson CCSG Radiation Oncology and Cancer Imaging Program (P30CA016672), the NIH/NCI Head and Neck Specialized Programs of Research Excellence (SPORE) Developmental Research Program Award (P50 CA097007) and the National Institute of Biomedical Imaging and Bioengineering (NIBIB) Research Education Program (R25EB025787). C.D. Fuller has also received direct industry grant support, speaking honoraria and travel funding from Elekta AB. L.V. van Dijk received/receives funding and salary support from the Dutch organization NWO ZonMw during the period of study execution via the Rubicon Individual career development grant.

## Declaration of Competing Interest

The authors declare that they have no known competing financial interests or personal relationships that could have appeared to influence the work reported in this paper.
