# Supplementary material for: Intensity standardization methods in magnetic resonance imaging of head and neck cancer
Source: Phys Imaging Radiat Oncol. 2021 Nov 20;20:88–93. doi: 10.1016/j.phro.2021.11.001 (PMC8607477; doi:10.1016/j.phro.2021.11.001)
Supplement: Supplementary data 1 [file mmc1.pdf]

Supplementary Material

Supplementary Data S1: SD NMI$_c$ Calculation Details

We used a simple and interpretable metric of comparison to quantify region-of-interest (ROI) intensity histogram overlap which can be applied before or after an intensity standardization procedure in a given cohort. The steps to calculate this metric are illustrated in Figure A1 and given below.

1. Calculate the mean of a given ROI intensity distribution for a patient (μ_p).
2. Divide μ_p by the range of ROI intensity distributions for the entire cohort ($s2_c - s1_c$), thereby "localizing" the mean for that patient with respect to the entire distribution of values. The resulting value is the cohort-level normalized mean intensity (NMI$_c$) for that ROI. $s1_c$ and $s2_c$ are set as the 2nd and 98th percentiles of ROI intensity distributions for the cohort, respectively, to remove any major outliers.
3. Calculate the NMI$_c$ for each patient in the cohort and subsequently measure the "spread" of these values for the entire cohort, i.e., the standard deviation of the NMI$_c$ (SD NMI$_c$) for that ROI.

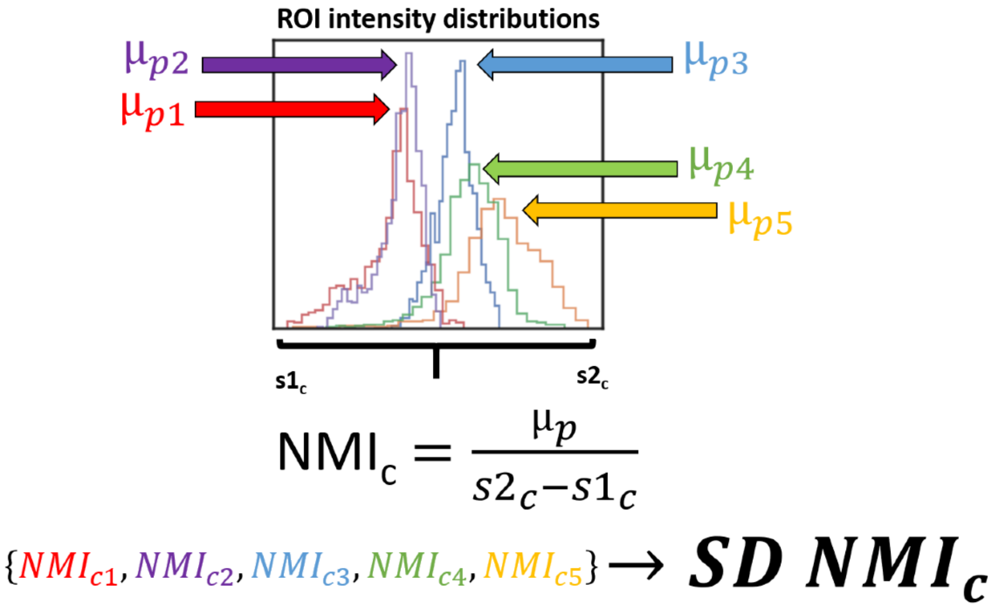

Supplementary Figure S1. A newly derived metric, the standard deviation of cohort-level normalized mean intensity (SD NMI$_c$), quantifies the region-of-interest (ROI) intensity distribution overlap. By measuring the variability of distributional mean values on a normalized scale, the SD NMI$_c$ provides a scale-invariant cohort-level measure of intensity standardization consistency. The SD NMI$_c$ is determined by calculating the standard deviation of a set of cohort-level normalized mean intensity (NMI$_c$) values. NMI$_c$ values are calculated by dividing the mean of a given ROI intensity distribution for a patient (μ_p) by the range of ROI intensity distributions for the entire cohort ($s2_c$ - $s1_c$).

SD NMI$_c$, as defined here, is an adapted metric from Nyul et al. [1] where instead of normalizing with respect to a set of intensity values for a given patient, we normalized with respect to a set

of intensity values for the entire cohort studied. The SD NMI_c is scale-invariant, so it can robustly measure variation across different standardization methods for a given ROI without introducing bias based on the scale of standardization.

References:

[1] Nyúl LG, Udupa JK. On standardizing the MR image intensity scale. Magn Reson Med An Off J Int Soc Magn Reson Med 1999;42:1072–81.

Supplementary Data S2: T1-weighted MRI Preliminary Analysis

In this appendix, we will use our workflow to analyze post-contrast Dixon T1-weighted images from a subset of 5 patients from the homogenous (HOM) cohort. The scanning characteristics of these T1-weighted images are shown in **Supplementary Table S1**. As in the T2-weighted HOM cohort analysis, all images were generated from the same scanner with identical acquisition parameters.

Supplementary Table S1. Homogeneous (HOM) cohort scanner characteristics for Dixon T1-weighted images. All five patients had the same scanner/acquisition parameters.

**Patient in cohort**	**1, 2, 3, 4, 5**
Manufacturer	Siemens
Manufacturer Model Name	Aera
Magnetic Field Strength (T)	1.50
Repetition Time (ms)	7.11
Echo Time (ms)	2.39
Echo Train Length	2
Flip Angle (°)	10
In-plane Resolution (mm)	1.00
Slice Thickness (mm)	1.00
Imaging Frequency (Hz)	63.67
Number Of Averages	2.00
Percent Sampling (%)	100
Pixel Bandwidth (Hz)	405.00
Acquisition Matrix	256x256

Of note, these Dixon T1-weighted sequences were water-suppressed, so water-containing structures (muscle, cerebrospinal fluid, cerebellum, etc.) are not clearly visible on imaging (**Supplementary Figure S2**) and were not included in the analysis. The left and right parotid glands were added as regions of interest since they were previously available. Moreover, while in the original analysis of T2-weighted images we opted to not include the cheek fat regions of interest in the statistical analysis since this could bias the results towards the *Fat* standardization method, we have chosen to include them here due to the limited number of structures visible on the Dixon images.

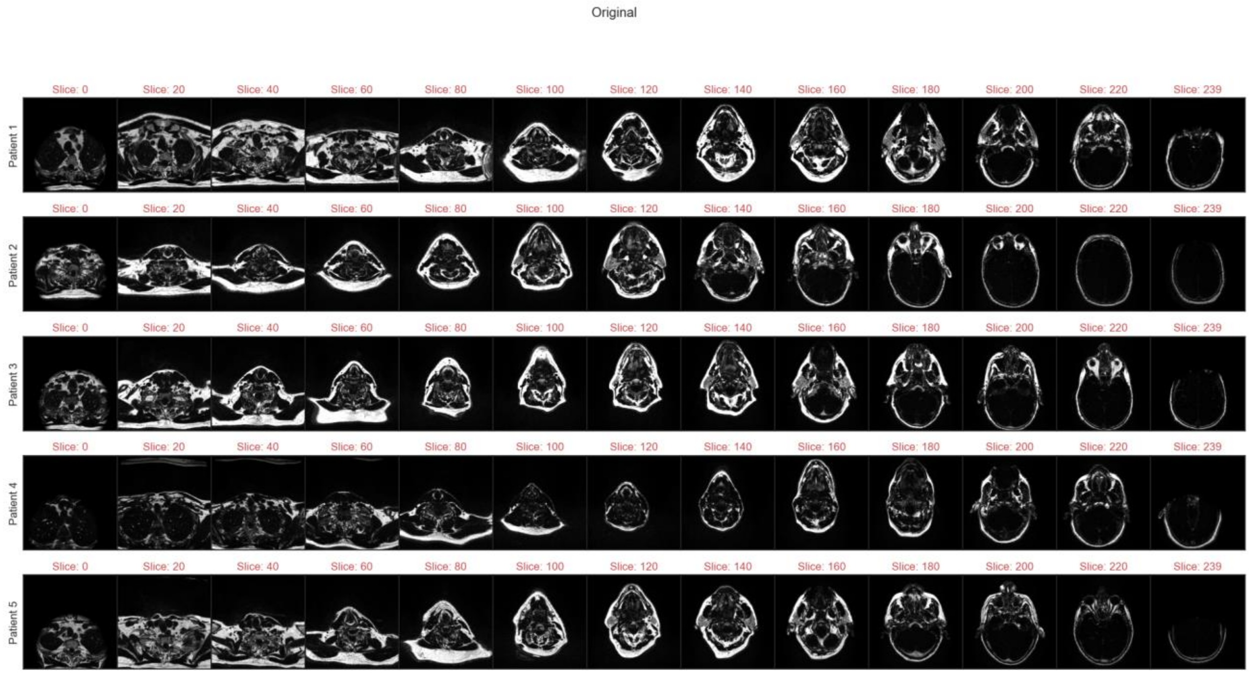

Supplementary Figure S2. Example Dixon T1-weighted Water Suppressed images for each patient in homogeneous (HOM) cohort corresponding to “Original” unstandardized scans. Images are at intervals of 1/13 total slices to visualize full field of view.

The heatmap of SD NMI_c values across all regions of interest and standardization methods for the Dixon T1-weighted images is shown in **Supplementary Figure S3**. The results are generally consistent with what is shown for the T2-weighted results of the HOM cohort in the main text. Specifically, there are generally minimal differences between the *Original* unstandardized images and the specific standardization methods. Moreover, the *Nyul* method seems to give the best quantitative results, which is also consistent with the main text. Upon application of the Friedman test, we found the p-value to be not significant (p=0.07), indicating no differences between standardization methods. Therefore, no post-hoc testing was employed. While these preliminary results are consistent with our main manuscript findings, further confirmatory work is needed before generalizing our observations to other MRI sequences.

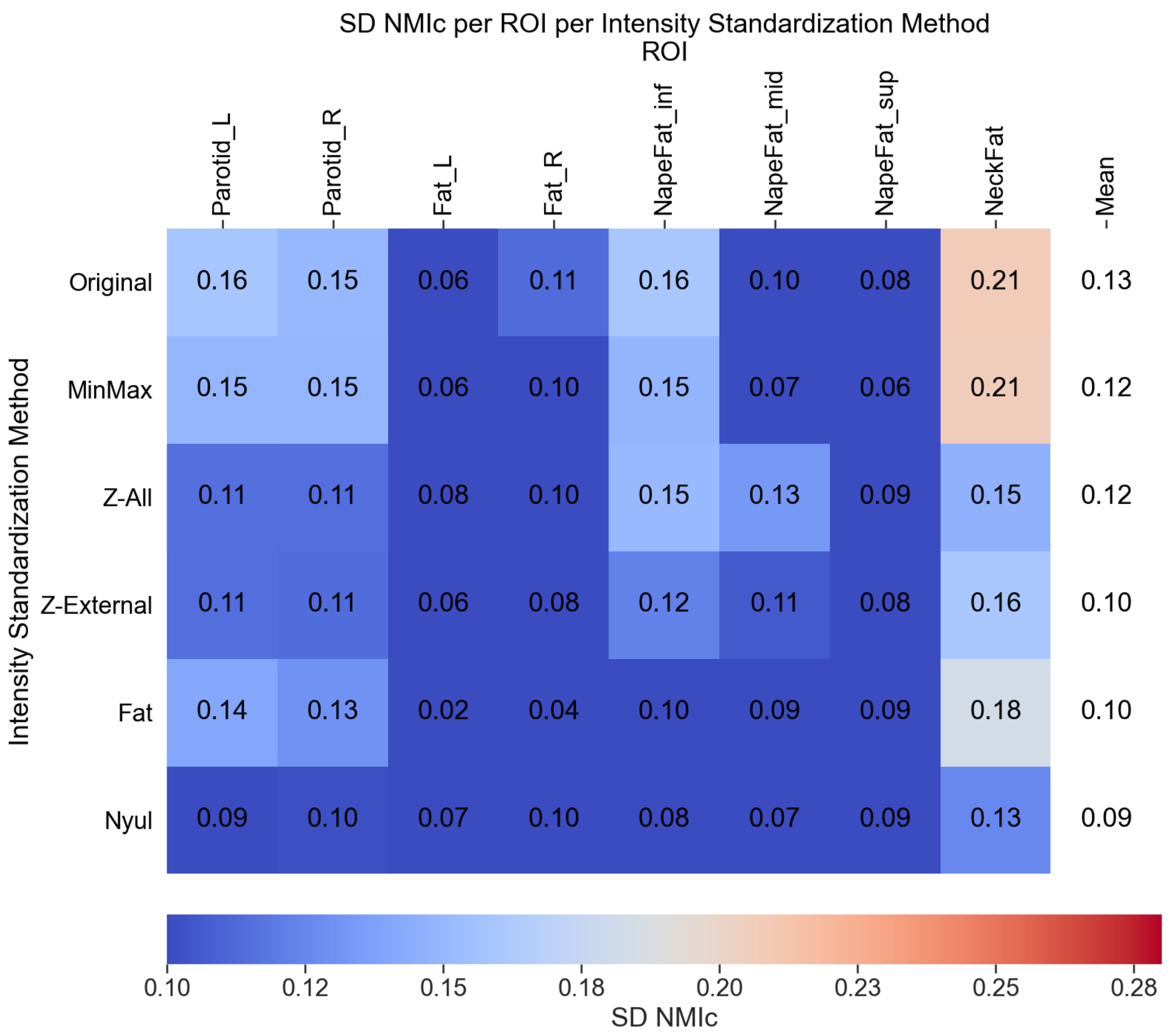

Supplementary Figure S3. Standard deviation of cohort-level normalized mean intensity (SD NMI_c) heatmaps per region of interest (ROI) with respect to standardization method for homogeneous (HOM) cohort Dixon T1-weighted Water Suppressed images. The resulting means across all ROIs for each method are shown in the rightmost column of the heatmap.

Supplementary Data S3: Additional Supplementary Figures

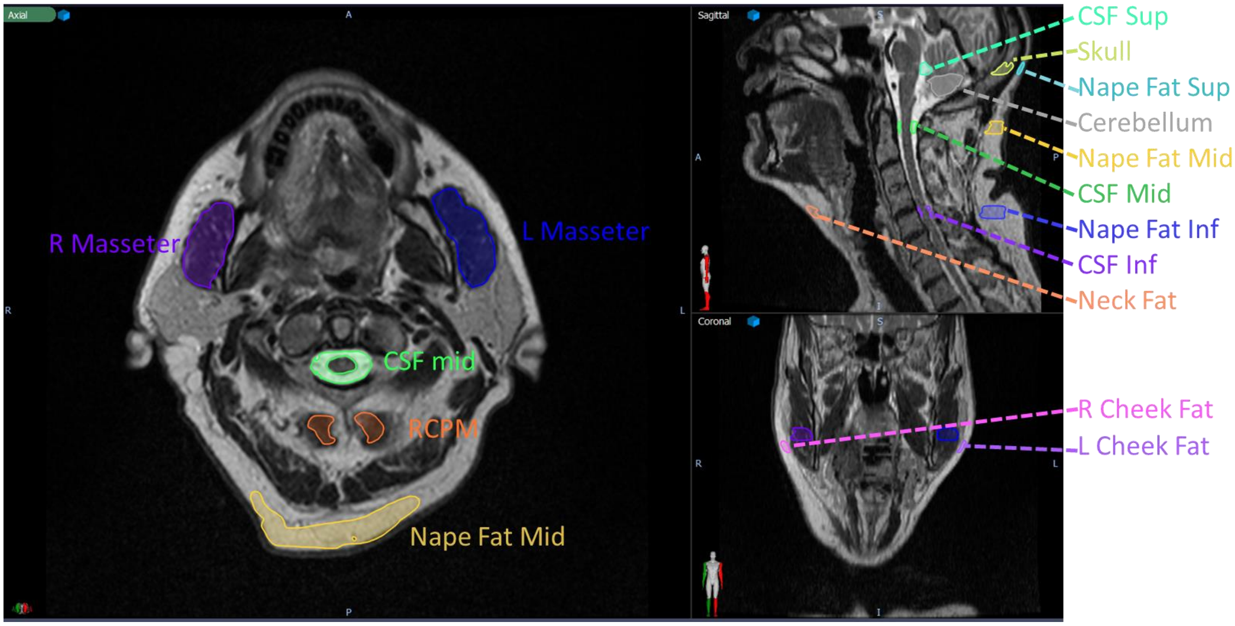

Supplementary Figure S4. Various regions of interest consisting of tissue types from different anatomical locations were contoured for all patients. All regions of interest were contoured for five slices in the same relative area for all patients. The T2-weighted image shown is an example from the homogeneous (HOM) cohort.

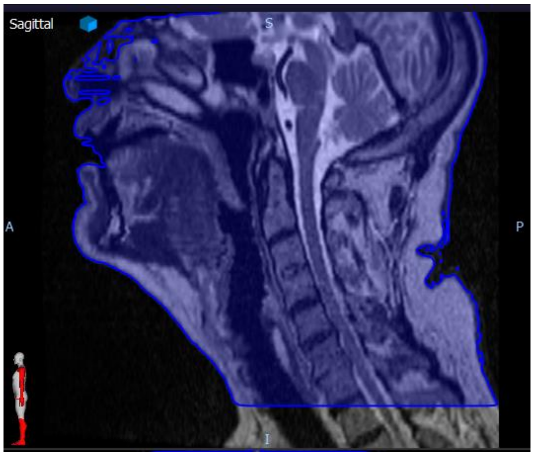

Supplementary Figure S5. Example of an external mask (blue outline) for a head and neck cancer patient in the homogeneous (HOM) cohort.

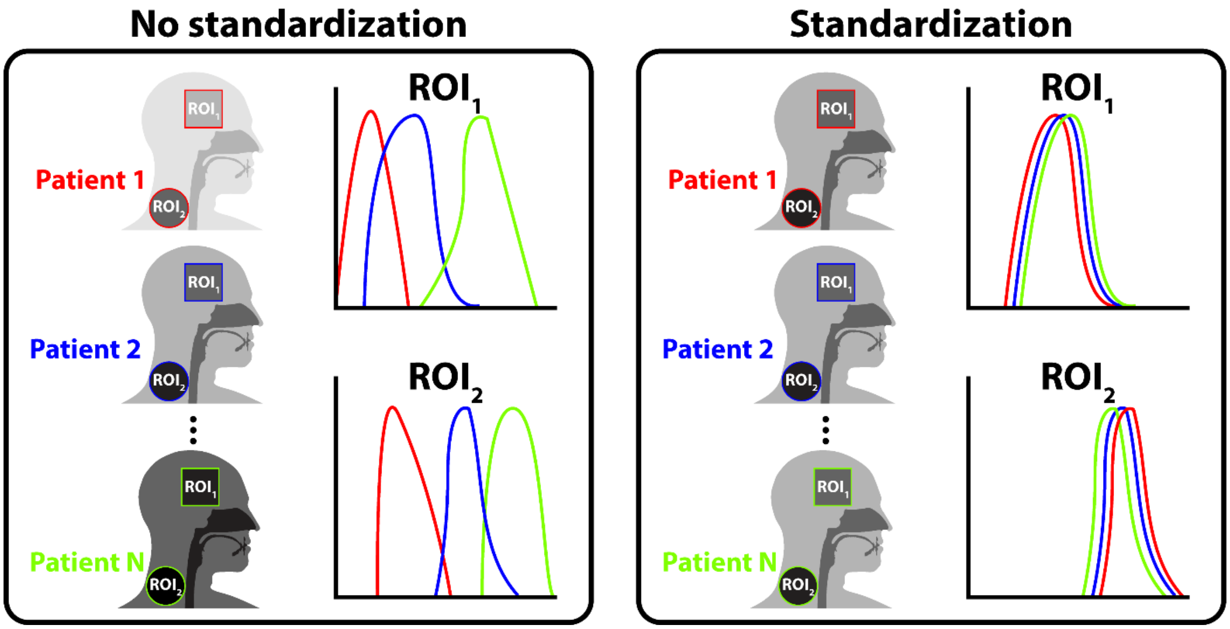

Supplementary Figure S6. Intensity standardization qualitatively improves nonpathological region of interest (ROI) intensity distribution consistency at the cohort level. Nonpathological ROIs corresponding to healthy tissues with assumed phenotypic similarity are not expected to vary between patients. Therefore, a proper intensity standardization aims to eliminate distributional differences between the same reference ROIs across patients.

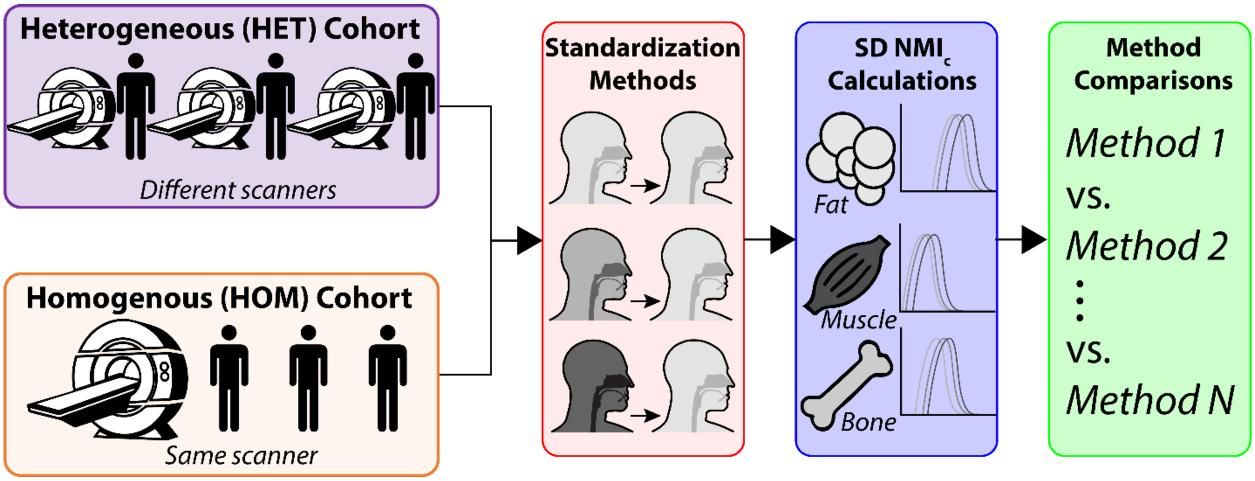

Supplementary Figure S7. Analysis workflow. Intensity standardization methods were applied to two separate cohorts of HNC patients who underwent MRI with either various scanners/acquisition parameters (HET cohort) or uniform scanners/acquisition parameters (HOM cohort) and used to calculate the standard deviation of cohort-level normalized mean intensity (SD NMI$_c$) for distinct healthy tissues. Intensity standardization methods were subsequently compared in each cohort through significance testing.

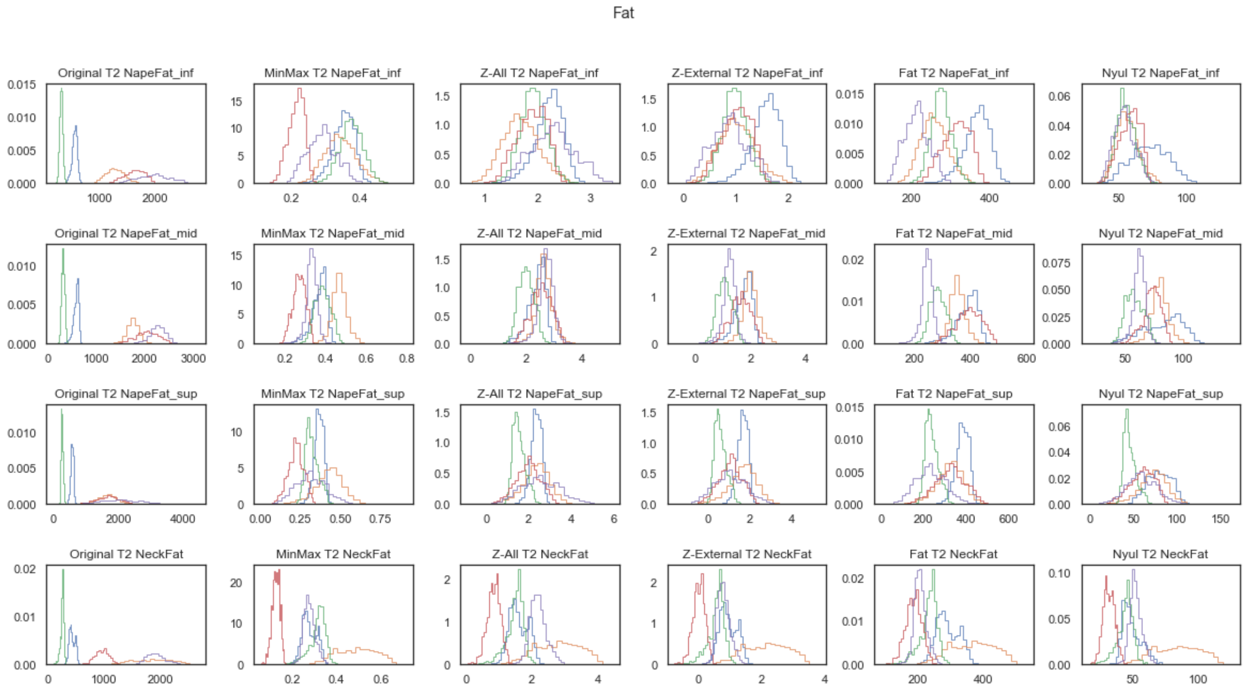

Supplementary Figure S8. T2-weighted MRI voxel intensity distributions in “Fat” regions of interest for a subset of 5 patients in the heterogeneous (HET) cohort. Different colored histograms correspond to different patients in cohort. Columns correspond to methods while rows correspond to regions of interest.

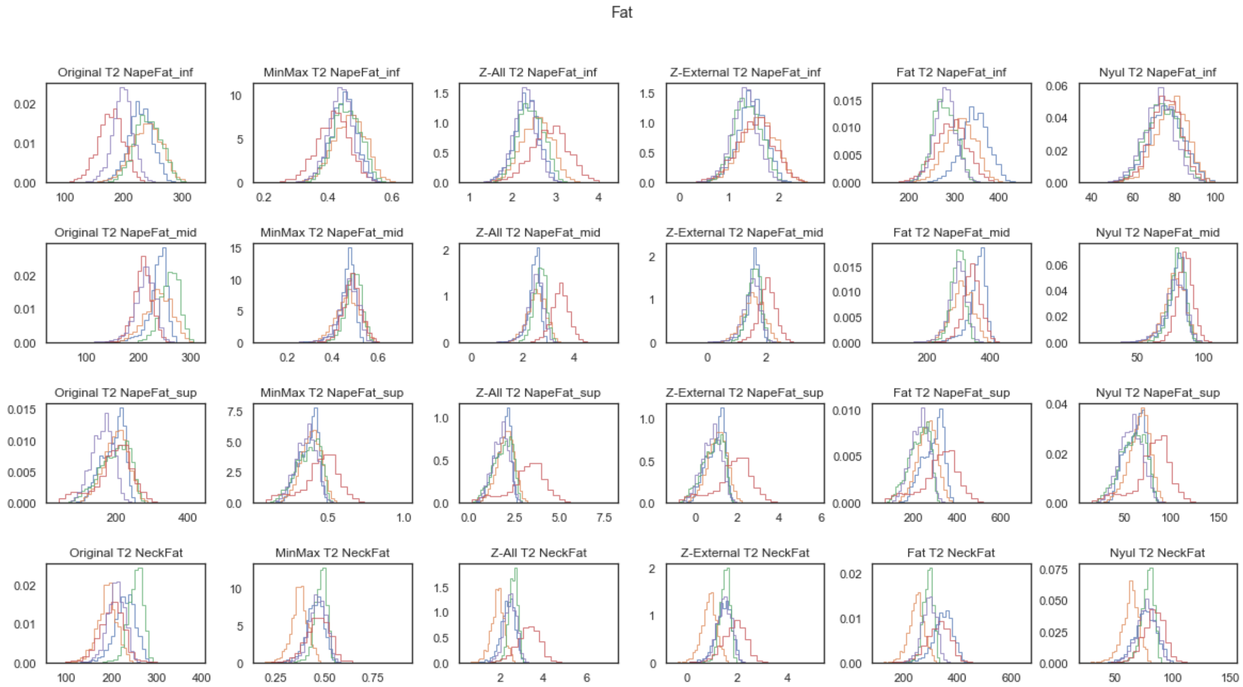

Supplementary Figure S9. T2-weighted MRI voxel intensity distributions in "Fat" regions of interest for a subset of 5 patients in the homogenous (HOM) cohort. Different colored histograms correspond to different patients in cohort. Columns correspond to methods while rows correspond to regions of interest.

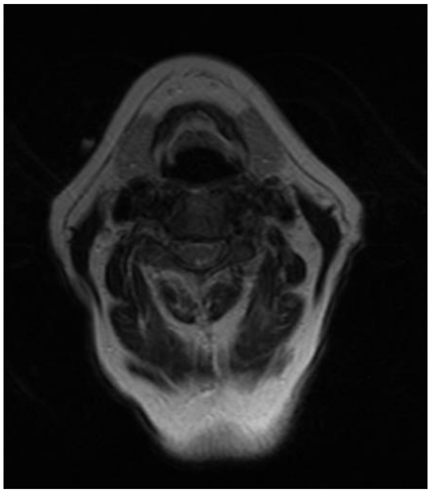

Supplementary Figure S10. An example of an MRI bias field signal present in a T2-weighted image from a patient from the heterogeneous (HET) cohort. Bias-field can be visualized in posterior-to-anterior direction.
